# The acceptability of compassion-focused therapy in clinical populations: a metasynthesis of the qualitative literature

**DOI:** 10.3389/fpsyt.2025.1400962

**Published:** 2025-02-27

**Authors:** Charlotte Garrett, Debbie M. Smith, Anja Wittkowski

**Affiliations:** ^1^ Division of Psychology and Mental Health, Faculty of Biology, Medicine and Health, School of Health Sciences, University of Manchester, Manchester, United Kingdom; ^2^ The Perinatal Mental Health & Parenting (PRIME) Research Unit, Greater Manchester Mental Health National Health Service (NHS) Foundation Trust, Manchester, United Kingdom; ^3^ Division of Psychology & Mental Health, Manchester Academic Health Science Centre, Manchester, United Kingdom

**Keywords:** compassionate, acceptability, psychological therapy, qualitative, self-compassion

## Abstract

**Background:**

Compassion-focused therapy (CFT) is a psychological intervention that is increasingly used in UK NHS services, either in an individual or a group format, with individuals experiencing psychological difficulties. Reviews of the quantitative evidence suggest that CFT effectively improves psychological well-being in various clinical groups. Participant experiences of group CFT in those with psychological difficulties have also been explored in several published qualitative and mixed-methods studies. Thus, the aim of this review was to further our understanding of the acceptability of group CFT, in relation to both the content of the intervention and its delivery, in order to help inform the future design and delivery of CFT in clinical services.

**Method:**

Following the Preferred Reporting Items for Systematic Review and Meta-Analysis guidelines, eight relevant databases were searched for terms associated with CFT and qualitative research. The methodological quality of included studies was appraised using the Critical Appraisal Skills Programme (CASP) screening tool. Findings were synthesised using thematic synthesis.

**Results:**

Twelve studies involving 106 participants with psychological difficulties met inclusion criteria. Five main themes were developed from the extracted data: 1) participants’ experiences prior to the intervention, 2) initial response to the idea of participation, 3) participants’ experiences of the intervention: aspects valued or considered beneficial, 4) valued outcomes of the intervention, and 5) the end of the intervention and moving forward.

**Conclusions:**

Findings indicated a high level of acceptability of group CFT and commonality of experiences across participants despite different clinical presentations. The crucial role played by facilitators and other group members to participant engagement and outcomes was highlighted, among other factors. Clinical and research implications of these findings are discussed.

## 1 Introduction

Compassion-Focused Therapy (CFT) is a psychotherapeutic intervention which aims to enhance psychological well-being by increasing an individual’s capacity for compassion towards themselves and others (1, 2). CFT is an evolution-informed biopsychosocial approach. It is an integrative, process-based therapy that draws on Buddhism, attachment theory, cognitive-behavioural theory, psychodynamic and humanistic theories, combined with insights from the field of neuroscience (2). As high levels of self-blame and criticism are a feature of many psychological disorders and difficulties (3), CFT can be used with individuals with a variety of clinical diagnoses and psychological difficulties (4–6). For example, CFT has been used successfully with individuals with personality disorders (7), eating disorders (8), bipolar disorder (9) and post-traumatic stress disorder (10). It has also been used in inpatient (11) as well as outpatient settings (7, 10, 12). CFT can be delivered individually or in a group format (4, 5). A variety of compassion-based interventions exist (13), but CFT, as developed by Gilbert (1, 2), is the most evaluated therapeutic approach to date (14) and is frequently used in NHS services as an intervention for mental health and/or psychological difficulties.

A key concept in CFT is that of the ‘tricky brain’ (2, 15). Our brains, Gilbert argues, were designed ‘for us and not by us’ by evolution, to help us survive (16). Our ‘old brain’ structures are responsible for more basic human motives (like avoiding harm and finding food), emotions (like anger, disgust, and anxiety) and behaviours (flight, fright, freeze) that are important for survival (16). Evolved structures of the human brain (e.g., the prefrontal cortex) are responsible for more sophisticated functions like planning, analysing, mentalising, and self-monitoring (15, 16). The ‘tricky brain’ in CFT describes the ways in which modern-day stressors (e.g., imagined failure) activate the threat system in a similar way as the kinds of external threats human beings encountered in early evolutionary times (e.g., the sight of a hungry lion). If enduring, these patterns of overactive threat-based thinking can lead to chronic emotional challenges (15–17).

In CFT, individuals are guided toward understanding these patterns through psychoeducation about three core components of our emotional-motivational functioning (15), referred to as the ‘threat’, ‘drive’ and ‘soothing-affiliative’ systems. These systems act together to facilitate survival by enabling us to detect and respond to potential threats and opportunities in the environment (what Gilbert calls the ‘threat’ and ‘drive’ systems) as well as promoting affiliation with the social group (the ‘soothing’ system) (2). The threat system, Gilbert argues, is necessarily the dominant system as it is focused on our immediate protection (2). It is responsible for flight, fright, freeze and appease responses and, when activated, this system gives rise to emotions such as anger, anxiety and disgust (2, 15–17). The drive system is energising and activating, motivating us to seek and obtain resources such as goods, sex, material possessions and social status (2). Emotions associated with activation in this system are excitement, pleasure, joy, sense of striving and achievement (2, 15). The soothing system is responsible for our propensity as human beings to care and protect, to connect with and soothe one another; tendencies that have also been key to our survival as a species (2, 15). When the soothing system is activated we feel emotions of calmness, contentment, warmth, connection, friendliness and kindness (2, 15).

Gilbert argues that a key cause of psychological distress and disorder in human beings is a relative imbalance in the activity of the three emotional-motivational systems (2, 15). Typically, this involves increased activation in the threat and sometimes the drive systems relative to the soothing-affiliative system (18). The activity of all of the emotional-motivational systems is impacted by an individual’s life experiences, particularly early ones, as well as, or in interaction with, the individual’s current social, cultural and political context (2, 15). For example, experiences of criticism, rejection and abuse in childhood, when individuals have not being offered care, protection, or been soothed by others when scared or upset, are likely to leave an individual with an underdeveloped soothing-affiliative system and an overreactive threat system, particularly in relation to social threat (2, 15). When these individuals are offered care and kindness, there may be difficulties for them in accessing it. If an individual’s early experience has been that love and care appeared conditional on achievement, they are likely to develop an overactive drive system (2). Overactivity in both threat and drive systems can manifest in a pattern of behaviour that has been referred to as ‘insecure striving’, whereby the individual, driven by the fear of social disapproval, is caught in a cycle in which they are continuously and relentlessly striving to achieve the next goal (19). This pattern of insecure striving has been found in the literature to have strong links with psychopathology (19). Attention then turns to learning about how the mind’s soothing system can be stimulated and developed to help them regulate their emotions and enhance their psychological wellbeing (20). This is done, in part, by helping individuals navigate and work through fears, blocks and resistances to giving or receiving compassion (21).

CFT interventions can be tailored to the specific needs of people with different clinical presentations who are experiencing various psychological difficulties (5). However, all CFT interventions involve two main components -a psychoeducational component and a practical, skills-based component (5). In the psychoeducational component, individuals are introduced to the concept of self-compassion and the evolutionary perspective on the mind that underpins CFT (4, 5). This includes discussion of the so-called ‘tricky’ brain, and the three emotional-motivational systems, and how their operation can give rise to the experience of suffering. The role of self-criticism, shame and self-blame in maintaining psychological distress is explained. A key message of CFT, which is implicit in the model, is drawn out and emphasised: our suffering as human beings is not our fault, but, rather, is the product of our evolved brains and personal histories. As such, suffering is also an experience that all human beings share and one toward which we can learn to be helpful.

An important component of CFT is Compassionate Mind Training (CMT) (20), the aim of which is to help individuals learn the skills required to develop the key attributes of compassion: care for well-being, sensitivity, distress tolerance, empathy and non-judgement (16, 20). As described by Leaviss and Uttley (22), the specific skills needed to develop these attributes are multi-sensory and common to other psychotherapies, and include compassionate reasoning, compassionate behaviour, compassionate imagery, compassionate feeling and compassionate sensations. Individuals are taught compassionate exercises to help them develop these skills which are practiced in session and between sessions as homework (5). Specific examples of these exercises include soothing rhythm breathing (a specific pattern of breathing designed to activate the soothing system), compassionate-self meditation (imagining oneself embodying the characteristics of an ideal compassionate self), and compassionate letter-writing (writing a compassionate letter to oneself when in a situation of difficulty or in response to self-critical thoughts) (20).

CFT can be delivered in an individual or group format. In NHS services in the UK, psychological interventions are often delivered in a group format for reasons of efficiency and cost-effectiveness. However, delivering psychological interventions in this format can also confer therapeutic benefits above and beyond the content of the particular intervention (23, 24). For example, it can help reduce social isolation and stigma associated with mental health conditions (23).

Several systematic reviews of the quantitative literature have examined the evidence base for compassion-based interventions. Although these reviews differ in focus, they provide evidence for the effectiveness of compassion-based interventions across a wide range of participant groups, including clinical groups, and participant outcomes (4, 5, 13, 22, 25). In their systematic review and meta-analysis of 22 randomised controlled trials (RCTs) of compassion-related therapies in clinical and sub-clinical groups, Wilson et al. (6) reported that, in comparison to control groups, compassion-based interventions produced greater improvements across all of the outcomes analysed, namely self-compassion (g = 0.52, 95% Cis [0.32, 0.71], anxiety (g = 0.46, 95% Cis [0.25, 0.66]) and depressive symptoms (g = 0.40, 95% Cis [0.23, 0.57]). Wilson et al. (6) included studies of all interventions which had the stated goal of directly or indirectly improving an individual’s level of self-compassion. This broad definition included mindfulness-based cognitive therapy and acceptance and commitment therapy, which are not necessarily identified as self-compassion interventions. Another relevant systematic review was conducted by Craig et al. (4) who examined the effectiveness of CFT in clinical populations by including controlled trials and observational studies alongside RCTs (n = 29). To be included in this review, study interventions had to cover what the authors considered to be core components of CFT (psychoeducation on the ‘tricky brain’ and three emotion-regulation systems, and compassion-building practices such as soothing rhythm breathing and compassionate imagery). The resulting review included studies of interventions, such as CFT, mindful self-compassion (MSC), compassion-based cultivation training (CBCT) and cognitively based compassion training (CBCT). Craig et al. (4) found that most included studies recorded gains on measures of mood and/or psychopathology for those receiving CFT, as well as in compassion (when reported). In all but eight of these 29 studies, CFT was delivered in a group format, with all these studies finding positive effects on study outcomes.

The most up to date systematic review of the quantitative literature on the effectiveness of CFT in clinical groups was conducted by Millard et al. (5), who included only RCTs and randomised pilot/feasibility studies and focused purely on CFT derived from the work of Paul Gilbert. Millard et al. (5) identified 15 studies. Within their 15 included studies, a group format was the most common method of intervention delivery (n = 8, 55.33%). Findings suggested that CFT was effective in improving a range of compassion-based outcomes and clinical symptomatology from baseline to post-intervention and compared to waitlist control, with small to moderate effect sizes for CFT on each of these outcomes. However, due to the small number of studies including an active control-group (n = 5), and the differences in intervention format and delivery between these studies, Millard et al. (5) could not conclude if CFT was more effective than other psychological interventions.

Although these reviews indicate that compassion-based interventions appear to be effective in improving outcomes in different participant groups, less is as yet known about the acceptability of this type of psychological intervention. In their review, in addition to effectiveness, Craig et al. (4) attempted to gauge the acceptability of CFT by examining quantitative data on attrition, service user satisfaction and compliance (when presented). Fifteen of the 29 included studies (51.72%) contained information on one or more of these variables. Attrition (reported in n = 13, 44.83% of the 29 studies) varied considerably between studies (from 0% to 52%), with an indication that attrition might be higher in those with more severe and complex mental health problems. All studies which reported service user satisfaction and compliance with the intervention (n = 15, 51.72% of studies) suggested that the intervention was acceptable and feasible. As Craig et al. (4) point out, the attrition rate reported across their included studies was somewhat lower than the average for drop-out from psychological interventions [30-50%, 26] which they suggest might potentially suggest a higher level of overall satisfaction with CFT compared to other psychological interventions, at least in clinical populations.

To date, no systematic review has explored acceptability indicators through the synthesis of participant accounts. However, an in depth understanding of the service user experience is recognised as being essential in the development of complex psychological interventions (27). Qualitative methodologies provide a means to access those experiences; findings often provide important insights into what aspects of an intervention and its delivery may need to be modified to best meet the needs of a particular client group, in a given context or setting in order to maximise patient engagement and satisfaction (28). There are a few studies that used qualitative methodology to gain insight into participant experiences of compassion-based interventions, some of which involve clinical groups [e.g., (7, 9, 10)]. Many of these studies use qualitative methodology alongside quantitative methodology as part of a mixed-methods evaluation or a pilot study (9, 29). These studies suggest that compassion-based interventions were experienced positively by participants/service users who considered the intervention content to be relevant and helpful, and who were able to identify tangible benefits of taking part which were valuable and meaningful to them.

Synthesising results across qualitative studies can help identify common themes for consideration, as well as identifying issues that may be particular to specific clinical groups (30). In line with the Medical Research Council (MRC)’s process evaluation framework (31), a systematic review and metasynthesis of the qualitative literature would inform the implementation of group compassion-based interventions and suggest intervention refinement that might be necessary to meet the needs of service users so that services can achieve maximum engagement and retention to ensure delivery is clinically and cost effective. Thus, the aim of this review was to explore participant experiences of CFT and to develop a more comprehensive and nuanced understanding of the acceptability of group CFT for individuals experiencing psychological difficulties by synthesising qualitative data, with a focus on the acceptability of intervention content and delivery.

## 2 Methods

The metasynthesis was conducted in line with the Preferred Reporting Items for Systematic Reviews and Meta-Analyses (PRISMA) guidelines (32). The protocol was registered with the International Prospective Register of Systematic Reviews (PROSPERO) in February 2022 (reference: CRD42022311248).

### 2.1 Search strategy

The search strategy, informed by the PICOS (Population, Intervention, Comparator, Outcome, Study Design) framework (33), was developed in consultation with a university librarian. Search terms for the intervention block were developed based on terms included in previous reviews of the literature on compassion-based interventions [e.g., (4, 5)]. A preliminary search indicated that terms associated with the intervention and the study design only were required to achieve appropriate sensitivity and specificity. Thus, these two blocks of search terms were included in the final search of eight databases relevant to this topic area: CINAHL Plus, EMBASE, MEDLINE, PsycARTICLES, PsycBOOKS, PsycINFO, PubMed and Web of Science. Databases were searched in April 2022 for articles published from inception that contained the terms outlined in **Table 1**. The search was updated in January 2024. Google Scholar and reference lists of included studies were also searched (34, 35).

**Table 1:** Search terms and limits.

	Block	Search terms & limits
1	Intervention	[(compassion or compassionate, or compassionate mind or compassion-focused or compassionate imagery) and (intervention or treatment or therapy* or training or exercise* or course or program*)]
2	Study methods/design	(qualitative or interview* or focus group* or mixed method* or IPA or Grounded Theory or Thematic Analys$ or narrative$).
3	1 AND 2	

Identified references were imported into EndNote (36). Duplicates were removed and titles, keywords and abstracts assessed for eligibility against the inclusion and exclusion criteria by the first author. The initial screening of titles and abstracts was carried out by the first author. A second independent reviewer screened 10% of the total number of studies for inclusion. Substantial agreement on inclusion/exclusion decisions was found between the first author and the independent reviewer (100%, kappa = 1.0) at the title/abstract screening phase. The first author reviewed the full text of studies that were not excluded during the screening stage. In the case of uncertainty, studies were discussed by all members of the research team and a decision was made jointly.

### 2.2 Inclusion and exclusion criteria

The PICOS framework (33) was also used to operationalise the inclusion and exclusion criteria, which are outlined in **Table 2**. Qualitative and mixed methods studies were included, provided it was possible to extract qualitative information from those studies. For the purposes of this review, we focused exclusively on CFT derived from the work of Paul Gilbert (2, 18, 37), comparable to Millard et al.’s review (5). We also decided to focus on interventions delivered in a group or primarily in a group format. The reasons for this were several. Firstly, it was considered that the service user experience of CFT delivered in a group format might be considerably different compared to CFT delivered individually. Secondly, previous reviews have shown that CFT is more commonly delivered in a group format. Thirdly, given that a group format has advantages in terms of cost-effectiveness and efficiency of delivery for services, concentrating on group CFT would produce the most clinically meaningful findings.

**Table 2:** Inclusion and exclusion criteria within the PICOS framework.

PICOS	Inclusion	Exclusion
**Population**	Adult participants experiencing psychological difficulties or displaying maladaptive coping behaviours indicative of underlying psychological difficulties (e.g. self-harm).	Non-adult samples not experiencing psychological difficulties. This includes: • Parents/carers of children diagnosed with a clinical physical/mental health condition. • Healthcare workers, school teachers or other professionals in healthcare, education or social care.
**Intervention**	Compassion Focused Therapy delivered in a group format, covering the primary components, which derive from the work of Paul Gilbert (2, 28, 29). These primary components include: ○ Psychoeducation such as on the concept of compassion, the three regulatory affect systems, ‘tricky brain’, fears of compassion, the role of shame and self-criticism ○ Exercises such as compassionate letter writing, compassionate attention, soothing rhythm breathing.	Other compassion-based interventions (e.g., compassion cultivation training, Mindful Self-Compassion). • Mindfulness-based interventions, whereby the focus is primarily on mindfulness rather than the core compassion therapy components as identified in the inclusion criteria. Studies that looked at CFT where there was significant variation from standard protocols such as culturally-adapted CFT (30). Compassion-focused therapy delivered in a 1:1 format.
**Comparison (Types of qualitative data collection and analysis)**	Recognised qualitative methods of data collection (e.g., focus groups, qualitative interviews) and analysis [e.g., interpretative phenomenological analysis [IPA; (31)], thematic analysis (32), and content analysis (33)].	Non-standard qualitative methods of data collection or analysis methods of analysis that were unreferenced (e.g., ‘a qualitative analysis was performed’ or ‘themes were extracted’ with no reference provided).
**Outcome (phenomenon of interest)**	Service user experiences and perceptions of CFT interventions.	Studies where service users/participants were asked to compare their experiences of CFT with other psychological interventions.
**Study Design**	Qualitative studies or mixed methods pilot/feasibility studies.	Studies using only quantitative methodologies. Grey literature including conference abstracts, reports, government documents.

### 2.3 Methodological quality and risk of bias assessment

The quality of included studies was assessed by one of the study authors (CG) using the 10-item Critical Appraisal Skills Programme (CASP) checklist for qualitative studies (38). To summarise quality ratings concisely and to provide a useful indicator for comparison, the items on the CASP checklist were attributed a numerical outcome (No = 0, Can’t Tell = 0.5, Yes = 1), resulting in a maximum total score of 10. The total CASP score for all papers was then used to categorise the methodological quality of the studies included in the review as either high (8–10), moderate (6–8) or low (<5). This approach has previously been used in other reviews and metasyntheses [e.g., (5, 39)]. An independent reviewer rated 25% of the papers. Decisions made by the first and second reviewers were identical for all three papers (100%, kappa = 1.0).

### 2.4 Data extraction and analysis

All text under the headings ‘*Results*’ or ‘*Findings*’, including quotations from participants, were extracted from the included papers into Microsoft Word and analysed using Thomas and Harden’s thematic synthesis approach (40). Author interpretations and themes from *Results* sections of included studies were also extracted to inform the analysis. Thomas and Harden’s (40) approach allows findings from multiple qualitative studies to be integrated via the identification of common themes across studies. As discussed by Barnett-Page and Thomas (41), this method can be used to generate novel insights concerning the appropriateness and acceptability of service provision, which in turn can then be used to inform both policy and clinical practice [e.g., (42, 43)].

The thematic analysis was conducted in three overlapping stages (40). Firstly, line-by-line coding was performed on the extracted text from each of the included studies. In the second stage of the analysis, codes referring to similar ideas were then grouped together to form descriptive themes. An inductive approach was taken to the development of descriptive themes, identifying similarities and patterns in service user experiences but also being mindful of dissimilarities or contradictions. In a final step analytical themes were developed from descriptive themes, which was achieved by considering the descriptive themes in the light of our review research aims, resulting in these new conceptual links and interpretations.

All stages of the analysis were undertaken by the first author (CG). The plausibility and coherence of themes was evaluated by another member of the research team independently reviewing the included studies (DS) and via scrutiny by the third author (AW). This process was followed to ensure codes and themes were appropriately derived from the data and potential bias was minimised.

### 2.5 Reflexivity statement

The design and conduct of this study were underpinned by a critical realistic epistemology (44). Within this approach, inferences can be made about psychosocial phenomena (in this case, participants’ experiences of compassion-focused therapy interventions), while acknowledging the contextual influences on these inferences, and that, although it is possible that these phenomena can exist independently of theory, meaning can also be constructed from the experiences reported within the included studies (44).

The authors were white European women. The first author was a trainee clinical psychologist with several years of experience as a researcher on projects related to the implementation of evidence-based psychological interventions for clinical groups in healthcare settings, and several years of clinical experience delivering psychological interventions to clinical groups, including compassion-based approaches. The second author was an academic psychologist specialising in health psychology research with an interest in compassion-based interventions. The third author was an academic and clinical psychologist with extensive experience of researching and implementing psychological interventions (including compassion-based interventions) in clinical groups. All authors have received training in CFT. As a team, we were conscious of evaluating the extracted data from a clinical research and mental health perspective. We were also conscious of the potential impact of our own interest in and experiences with CFT on our interpretation of the data. To minimise the potential for bias, we employed several techniques recommended in available guidance (45), including a reflective diary, research team discussions and ensured that the research process was rigorous and transparent.

## 3 Results

### 3.1 Characteristics of included studies

**Figure 1:**
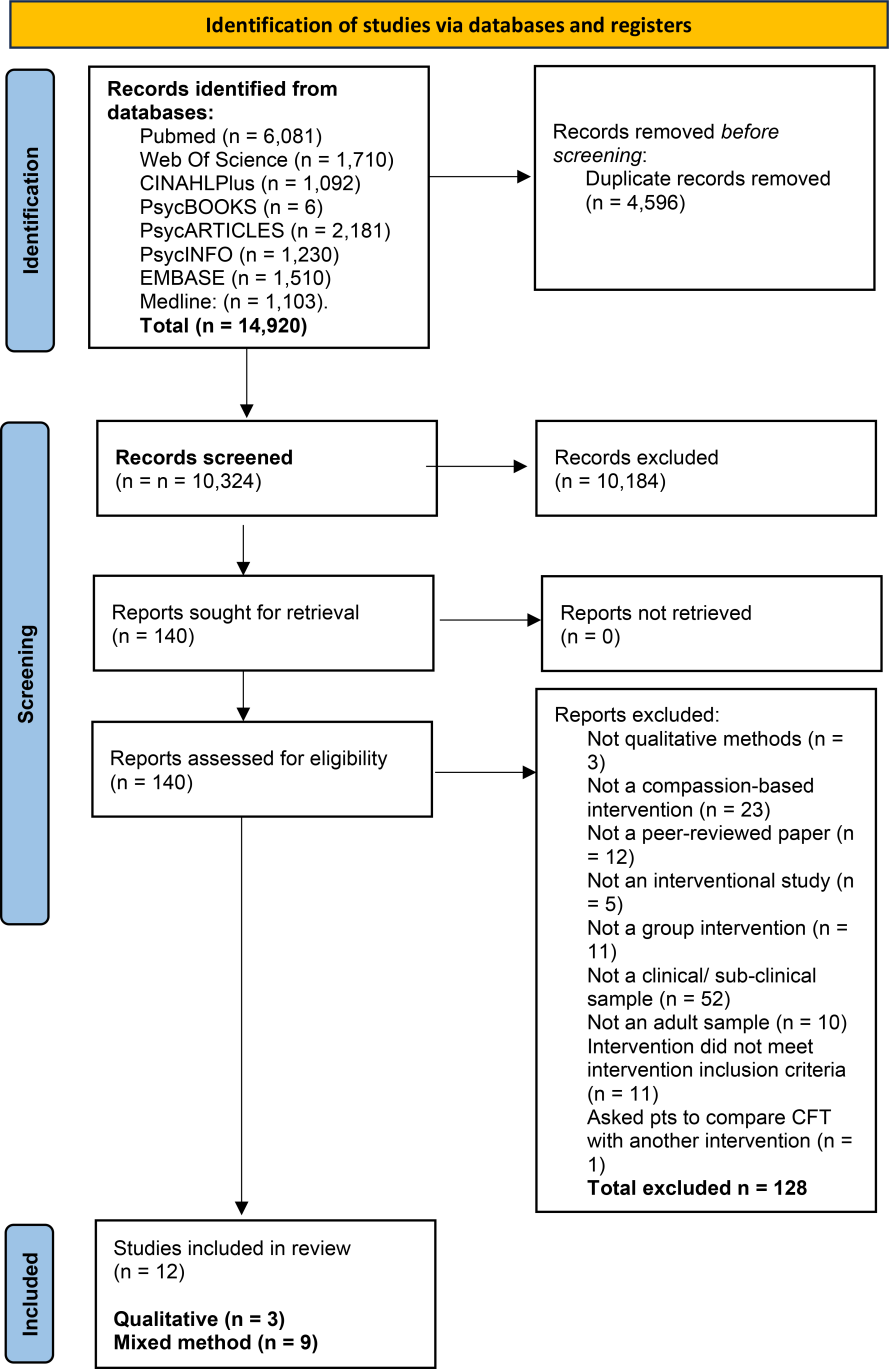
PRISMA diagram of search strategy.

**Figure 1** presents an outline of the search process based on PRISMA guidelines (32).

Twelve studies, conducted in three countries (the UK, Republic of Ireland and New Zealand) between 2012 and 2022, were identified and synthesised (see **Table 3** for details). They reported on the experiences of 106 individuals who had participated in group CFT interventions. Included studies evaluated CFT in individuals with a broad range of psychological symptoms and difficulties, including depression, anxiety, bipolar affective disorder, schizophrenia, complex trauma/PTSD, eating disorders, and personality disorders. Other samples included individuals with mood difficulties who also engaged in self-harm, individuals struggling psychologically with physical and/or neurological health issues (persistent pain and acquired brain injury) and individuals with intellectual disability who were experiencing low mood, high self-criticism and shame. Two of the 12 studies (11, 46) were undertaken with mixed samples which included individuals experiencing various psychological symptoms or with differing psychiatric diagnoses. Most studies (n = 10) were more homogeneous with regards to their sample’s clinical presentation. Most studies had small sample sizes (ranging from 3-11 participants) except for one study (12) which had a sample size of 31 participants.

**Table 3:** Characteristics of included studies presented in chronological order.

	Study: authors, year, location	Design/study type	Study aim(s)	Participants	Intervention	Data collection*	Method of analysis	Main themes (Author Identified)
1	Altavilla and Strudwick (2022) (46) UK	Mixed methods study	To evaluate and provide recommendations to improve the effectiveness of an age inclusive CFT group in secondary mental health services for individuals experiencing a range of mental health difficulties.	Convenience sample (n = 6) of the 23 participants who had completed one of four CFT groups run by the service. 5 participants were female and one was male. Three were considered working age range (18-64 yrs), and three were considered older adult (over 65 yrs). All were white-British with English as their first language. All were under secondary mental health services (either working age or older adult) and experienced a range of psychological difficulties. They had also been identified as having long-standing struggles with self-criticism/self-blame. Participants were excluded from taking part in the group if they had a significant cognitive impairment, were currently experiencing psychosis, or if they were substance-dependent and their use of substances was likely to impact their ability to engage.	Age-inclusive CFT group facilitated by two experienced clinical psychologists, trained and with experience working in a CFT approach. Supervision from a CP experienced in working within a CFT model. The group programme was developed by facilitators based on existing CFT group literature and published protocols (18, 47, 48). N = 6-10 participants per group. Group ran for 20 sessions, mainly on a weekly basis. Each session lasted 2 ½ hours with a coffee break. Sessions organised by theme for consistency but delivered flexibly to respond to the needs of group members. At the halfway point, each group member was invited to meet with a facilitator for 30 min to work on an individualised formulation. They were also invited to another 30-min individual session at the end of the group programme to review their overall experience of the group and plan for next steps.	Qualitative data: Semi-structured interview Quantitative data: Self-report measures (completed pre, mid and post- intervention & at 3 mo f-up): Self-Compassion Scale (SCS), Mindful Attention Awareness Scale (MAAS), CORE-34 and Depression Anxiety and Stress Scale (DASS)	Thematic analysis (49)	- Connection with others - The experience of diverse age - Group as a secure space
2	Gilbert et al. (2022) (9) UK	Mixed methods feasibility and acceptability study	(To examine the patient experience and feasibility of a 12 module CFT group tailored for individuals with a diagnosis of bipolar disorder.	N= 10 service users of a specialist bipolar service with a clinical diagnosis of bipolar affective disorder, relatively stable in mood at time of participation in the study. Not all the participants took part in every focus group.	Group CFT for bipolar disorder based on CFT manual in preparation^1^ with some adjustments for prior knowledge of participants. 12 sessions initially delivered over 14 weeks, a 20-week rest period and then another 13 sessions over 13 weeks. Delivered by 2 clinical psychologists, both trained and one with extensive experience in CFT and receiving regular supervision from Paul Gilbert.	Qualitative- Focus groups Quantitative data: Self-report measures: DASS, The Hospital Anxiety and Depression Scale (HADS), The Experiences Questionnaire- Decentering subscale (EQ), Positive Affect and Negative Affect Scale (PANAS), Three Types of Positive Affect Scale, Forms of Self-Criticism/Self-Reassuring Scale (FSCRS), Social Comparison Scale, Social Safeness and Pleasure Scale (SSPS), Compassion Engagement and Action Scale. Quantitative-Heart Rate Variability (HRV), measured using Biopack software in response to a series of imagined scenarios presented to participants ( relating to social rank, attachment, competition) interspersed with neutral scenarios.	Thematic analysis (49)	- Understanding and utilising the evolutionary model - Experiences of the degree of helpfulness of the CMT exercises. - Using CFT to understand and address self-criticism - General experience of the therapeutic process and impact on managing moods and emotions; - Going forward & suggestions for the future.
3	Maynard et al. (2023) (12) New Zealand	Qualitative study	To provide a more detailed understanding of participants’ experiences of change within the CFT groups, in relation to their experiences of self-forgiveness and psychological health.	N = 31 users of a community mental health service, described by authors as suffering from mild to moderate depressive symptoms and with low risk. Mean age of sample was 42.6 yrs (range 18-63 yrs). 39% male, 61% female. 29.3% single, 31.7% married, 28.8% divorced, 12.2% de facto relationship. 80.5% classified themselves as NZ/European, 12.2% as Maori, 2.4% as Pacific Peoples, 2.4% as either Latin American, Middle-Eastern/African origin, remaining 4.8% as another ethnic group.	12-week CFT intervention based on the True Strength protocol for managing difficult emotions (12). Duration of each session was 2 hours with a 15-minute break. Sessions led by a consultant clinical psychologist.	Focus groups	Thematic analysis (50) with an inductive approach to coding	- Becoming self-compassionate and self-forgiving. - The CFT group was beneficial.
4	Ashfield et al. (2021) (10) UK	Qualitative study	To investigate the mechanisms of change at an individual and group level for individuals completing a CFT-based intervention for individuals with complex PTSD	N = 11 service users with a diagnosis of PTSD attending a specialist PTSD service	Group CFT intervention for trauma based on CFT (1, 47, 51). Total number of session and intervention length not specified. Professional background and training of those delivering the intervention not specified. Supervision arrangements also not specified.	Interviews	Constructivist grounded theory (52)-based analysis of interview data	**Overall explanatory model:** An ongoing journey of change **Themes:** - Experiences before the group - Overcoming barriers and readiness for change - The change process
5	Goad and Parker (2021) (53) UK	Mixed methods study	To evaluate a CFT group intervention for people with intellectual disability experiencing low mood, high self-criticism, and feelings of shame	N = 6, service users with a diagnosed (mild) ID receiving support from an NHS Community ID service for difficulties with low mood, high self-criticism and shame. One participant also had diagnosed autism, another also had diagnosed autism and Attention-Deficit Disorder (ASD).	Extended version (11 sessions) of the 6-session group CFT intervention developed by Clapton et al. (54) for individuals with ID Delivered by a senior clinical psychologist with a high level of expertise and experience in delivering CFT. Supervision arrangements (if any) not specified.	Qualitative data: Focus groups Quantitative data: Self-report measures- CORE-LD, Adapted Social Comparisons Scale and the Self-Compassion Scale-Short Form	Inductive thematic analysis (49)	***Focus group 1:*** - Feedback obtained*;* - Developing compassion for self and others - Managing emotions - Developing connections ***Focus group 2:*** - Compassion to self - Compassion to others - Developing connection - Obtaining feedback
6	Raynor et al. (2022) (29) UK	Mixed methods evaluation	(Of the qualitative component) To explore the impact of a ‘compassion-focused cognitive behavioural therapy group’ for people that self-harm. To provide a detailed exploration of the experiences of the participants in the psychotherapy group.	N=3 individuals (n = 3) aged 16+ who had self-harmed more than three times in the last year, residing in the community, recruited from accident and emergency, universities and further education colleges, mental health and other local charities.	12 session psychotherapy group for people that self-harm integrating CFT (55) and CBT for self-harm (56) developed by four of the study authors. Delivered by behavioural psychotherapist, mental health nurse and a CBT therapist. Supervision provided by the first author (BABCP-accredited psychotherapist, mental health nurse and integrative counsellor Psychotherapist).	Qualitative data: Focus group Quantitative data: Self-report questionnaires: Patient Health Questionnaire-9 (PHQ-9), the General Anxiety Disorder-7 (GAD-7), the Self-Compassion Scale, (SCS), Cognitions of Self-Injurious Behaviour Scale completed at first and final sessions, then 3 month follow up.	IPA (57)	- The secret’s out! Openness and honesty - Care without fear: calm acceptance, - Skills not spills - We’re all in it together (acceptance) - Compassion, not competition or comparison - Fear of ‘flying solo’
7	Gooding et al. (2020) (58) UK	Mixed methods evaluation	To explore the effectiveness of a 12-week CFT group intervention for people with persistent pain in a clinical setting considering group and individual change processes.	Adult service users (N = 4) with persistent pain attending an NHS pain management service.3 participants were male, 1 female, aged between 47 and 76 years. Participants scored in the clinically severe ranges for both depression and anxiety on the DASS-21 and in the moderate range for stress.	12 session CFT group delivered over 12 weeks. Each session lasted two hours. This version of group CFT was based on Gilbert (1) and was adapted for the client group by clinical psychologists within the pain service where the intervention was being delivered, two of whom also facilitated the sessions. Any supervision arrangements in place were not described.	Qualitative data: Semi-structured interviews Quantitative data: Self-report questionnaires: DASS-21, The Forms of Self-Criticising/Attacking and Self-Reassuring Scale (FSCRS), The Chronic Pain Acceptance Questionnaire (CPAQ), The Pain Disability Index (PDI).	IPA (57)	- The immense impact of pain on daily life - Meaning of connection and belonging in the group - Engaging with the emotions connected to the pain experience - Recognising the process of change in the group - Applying learning from the group.
8	Mullen et al. (2020) (59) Republic of Ireland	Qualitative study	To explore service users’ experiences of attending a group CFT intervention for eating disorders as well as any possible changes in patterns of relating to self and others.	*N=* 9 service users meeting diagnostic criteria for eating disorder attending an outpatient clinic.	Group CFT intervention for eating disorders [CFT-E2, 8]. This intervention combines CFT with standardised CBT approaches to eating disorders [CBT-E, (60)]. The intervention comprises 24 sessions, 20 of which are delivered in a group format, 3 are individual review sessions and 1 is a friends and family session. Group sessions are between ½ a day and a full day in length. The intervention was delivered by two clinical psychologists. Supervision arrangements not specified.	Semi-structured interviews	Thematic analysis (49) and relational analysis (61).	- Flow of compassion and knowledge - Sharing, connecting, and belonging - Hope and trust - Structure and accountability - Strength, struggle and practice - Managing dilemmas
9	Clapton et al. (2018) (54) UK	Mixed methods acceptability and feasibility	To preliminarily investigate and explore whether a CFT group intervention is feasible and acceptable for adults with ID who have concurrent mental health issues.	N = 7 service users with an ID receiving support from NHS Community Learning Disabilities Teams. Service users identified as experiencing significant psychological distress (≥13 on the relevant index of the Psychological Therapies Outcome Scale for IDs (62), and significant self-criticism.	Brief (6-session) group CFT intervention adapted for the client group by the first author (Clapton) from existing group-CFT interventions in the research literature and based on Gilbert et al. (2, 55). Each session lasts for 90 minutes. Time over which 6 sessions were delivered not specified. Group facilitated by a clinical psychologist with extensive training and experience with CFT who attends regular group supervision with Paul Gilbert. Group 1 was co-facilitated by a senior clinical psychologist, group 2 by a trainee clinical psychologist (both less experienced with CFT approaches).	Qualitative data: Focus groups Quantitative data: Self-report measures: i) For inclusion in the study- Psychological distress index of the Psychological Therapies Outcome Scale for IDs (PTOS-ID); ii) Pre and post intervention measures (completed pre-intervention and at 2 and 4 weeks post-intervention)- Self-Compassion Scale-Short Form (SCS-SF), The Psychological Therapy Outcome Scale for Intellectual Disabilities (PTOS-ID),The Adapted Social Comparisons Scale (ASCS), CFT-ID session feasibility and acceptability measure (bespoke measure).	Thematic analysis (49)	- ‘*It’s like … you’re not on your own’-* Experiences of the group intervention and process - *‘It’s hard to be kind to yourself when you’re always used to not being kind to yourself’ –* Fears, blocks, and resistances to compassion. - ‘*Looking at yourself from the inside’-* Changes in relating to self, other and life experiences.
10	Ashworth et al. (2015) (63) UK	Mixed methods feasibility and acceptability study	To assess the feasibility, safety, and potential value of CFT for ABI patients with emotional difficulties	N = 7 service users with acquired brain injury and mental health difficulties attending a neurorehabilitation unit as outpatients.	CFT (17)-informed intervention developed and tailored for the client group and setting by the study’s first author (Ashworth) who had completed training in CFT and received monthly supervision from Paul Gilbert during the initial development of the intervention. CFT intervention (entitled ‘mood group’) delivered as part of a holistic neurorehabilitation program for individuals who had experienced ABI. Intervention delivered by three clinical psychologists who had all attended Paul Gilbert’s 3-day training in CFT. All received supervision from another qualified clinical psychologist.	Qualitative data: Semi-structured interviews Quantitative data: Self-report questionnaires (collected pre and post-intervention and at 3 month follow-up): HADS, FSCRS	IPA (57)	- Psychological difficulties - Developing trust and finding safeness - A new approach
11	Heriot-Maitland et al. (2014) (11) UK	Mixed methods feasibility and acceptability study/service evaluation	To examine the acceptability and feasibility of providing a CFT-group intervention adapted for the inpatient environment.	*N = 4* service users in an NHS acute inpatient psychiatric unit, most common diagnoses- schizophrenia, schizoaffective disorder, bipolar affective disorder, personality disorder, depression and anxiety.	CFT-informed (17) brief (4 session) group intervention adapted specifically for inpatient settings by study authors. Each session lasted 60 minutes and the intervention was delivered over 4 weeks. Intervention delivered by a clinical psychologist with training and experience in CFT who received regular supervision from a specialist CFT practitioner. A member of nursing staff from the ward or trainee clinical psychologist assisted with the delivery.	Qualitative data: Semi-structured interviews Quantitative data: Self-report measures: (Completed pre and post-session) Distress and calmness scales, (Completed post-session) Understanding and helpfulness ratings (bespoke 6-point measure).	Thematic analysis (49)	- Common humanity and affiliative relating - Understanding compassion - Activating of positive affect - Experiences of the group
12	Lucre and Corten (2013) (7) UK	Mixed methods pilot study	To evaluate the worth/value of a newly developed CFT groupwork programme for people with PD.	N = 8 service users with a Personality Disorder diagnosis (confirmed by a senior clinician in the service trained in the administration of the International PD Examination (a diagnostic instrument for PD) who also regarded themselves as ‘self-critical’ under the care of NHS secondary care services and referred to a specialist PD therapy service.	16-session group CFT intervention delivered over 16 weeks (length of session not specified). Version of CFT was based on Gilbert (1) and adapted for the clinical group by the study authors. The intervention was delivered by an accredited cognitive- behavioural psychotherapist and a ‘band 4 group facilitator’. These group facilitators had received training and attended monthly supervision from Paul Gilbert for the duration of the intervention.	Qualitative data: Verbal and written feedback from participants Quantitative data: Self-report measures: Social Comparison Scale (SCS), Submissive Behaviour Scale (SBS), The Other as Shamer Scale (OAS), FSCRS, DASS-21, CORE-34.	Content analysis (64)	- Taking responsibility for one’s thoughts and actions - The comfort of shared group experiences - Fear of compassion - Awareness of self-criticism and addressing it with assertive action

*1*ABI, Acquired Brain Injury; ADD, Attention Deficit Disorder; BABCP, British Association for Behavioural and Cognitive Psychotherapies; CBT-E, Cognitive Behavioural Therapy for Eating Disorders; CFT, Compassion-Focused Therapy; CMT, Compassionate Mind Training; CP, Clinical Psychologist; DASS-21, the Depression Anxiety and Stress Scale- 21 items; ID, Intellectual Disability; IPA, Interpretive Phenomenological Analysis; PD, Personality Disorder; PTSD, Post-Traumatic Stress Disorder; NZ, New Zealand; UK, United Kingdom.

*2*For further details of/references for questionnaire measures, please consult the original papers.

*Please see author reference for questionnaire details.

All but one of the 12 studies were undertaken in healthcare settings (see **Table 3**). The remaining study was conducted at a university campus (29). One study was undertaken in an inpatient mental health setting (11). Others were undertaken in outpatient settings, either in community mental health (*n* = 7), neurorehabilitation (*n* = 1) or learning disability services (*n* = 2).

Most of the included studies (*n* = 9) were mixed methods (qualitative and quantitative) evaluations of CFT interventions; only three studies (8, 10, 12) used qualitative methods exclusively. Qualitative data were derived from interviews (*n* = 6), focus groups (*n* = 5) and verbal and written feedback from participants (*n* = 1). Most studies used thematic analysis (*n* = 7), three studies used IPA, one used grounded-theory and the final study used content analysis (see **Table 3**).

### 3.2 Methodological quality of included studies

The methodological quality of all 12 studies was assessed as being high. However, only four of the studies included information that confirmed that they had adequately considered the researcher-participant relationship (critical examination of their own role and potential bias in the formulation of research questions and data collection) (10, 12, 46, 58). An example of a study that met criteria for this aspect of study quality was Ashfield et al. (10), who made explicit reference to having considered the subjectivity of the research process and influence of the researchers’ personal and professional experiences in their analysis, and specific methods that they had employed over the course of the research to ensure the credibility of their eventual research findings (e.g., reflexive interviews with researchers and reflective diaries for researchers, methods of constant comparison, formal and peer supervision for researchers and consultation with participants during the analytic process).

It was not possible to determine if the data analysis had been sufficiently rigorous in two studies (7, 29). In another two studies (11, 63), it was unclear if the recruitment strategy was appropriate to the aims of the research. The studies by Ashfield et al. (10), Gooding et al. (58), Maynard et al. (12), and Altavilla and Strudwick (46) received the highest possible rating for quality (10 points). The methodological quality of included studies is detailed in **Table 4**.

**Table 4:** Methodological quality assessment of the 12 included studies.

	Authors and publication year	1. Was there a clear statement of the aims of the research?	2. Is a qualitative methodology appropriate?	3. Was the research design appropriate to address the aims of the research?	4. Was the recruitment strategy appropriate to the aims of the research?	5. Was the data collected in a way that addressed the research issue?	6. Has the relationship between researcher and participants been adequately considered?	7. Have ethical issues been taken into consideration?	8. Was the data analysis sufficiently rigorous?	9. Is there a clear statement of findings?	10. Was the research valuable?	Total score (max score = 10)
**1**	Gilbert et al. (2022) (9)	Yes (1)	Yes (1)	Yes (1)	Yes (1)	Yes (1)	No (0)	Yes (1)	Yes (1)	Can’t tell (0.5)	Yes (1)	High (9)
**2**	Ashfield et al. (2021) (10)	Yes (1)	Yes (1)	Yes (1)	Yes (1)	Yes (1)	Yes (1)	Yes (1)	Yes (1)	Yes (1)	Yes (1)	High (9)
**3**	Goad and Parker (2021) (53)	Yes (1)	Yes (1)	Yes (1)	Yes (1)	Yes (1)	Can’t tell (0.5)	Yes (1)	Yes (1)	Yes (1)	Yes (1)	High (9.5)
**4**	Raynor et al. (2022) (29)	Yes (1)	Yes (1)	Yes (1)	Yes (1)	Yes (1)	Can’t tell (0.5)	Yes (1)	Can’t tell (0.5)	Yes (1)	Yes (1)	High (9)
**5**	Gooding et al. (2020) (58)	Yes (1)	Yes (1)	Yes (1)	Yes (1)	Yes (1)	Yes (1)	Yes (1)	Yes (1)	Yes (1)	Yes (1)	High (10)
**6**	Mullen et al. (2020) (59)	Yes (1)	Yes (1)	Yes (1)	Yes (1)	Yes (1)	Can’t tell (0.5)	Yes (1)	Yes (1)	Yes (1)	Yes (1)	High (9.5)
**7**	Clapton et al. (2018) (54)	Yes (1)	Yes (1)	Yes (1)	Yes (1)	Yes (1)	Can’t tell (0.5)	Yes (1)	Yes (1)	Yes (1)	Yes (1)	High (9.5)
**8**	Ashworth et al. (2015) (63)	Yes (1)	Yes (1)	Yes (1)	Can’t tell (0.5)	Yes (1)	No (0)	Yes (1)	Yes (1)	Yes (1)	Yes (1)	High (8.5)
**9**	Heriot-Maitland et al. (2014) (11)	Yes (1)	Yes (1)	Yes (1)	Can’t tell (0.5)	Yes (1)	Can’t tell (0.5)	Yes (1)	Yes (1)	Yes (1)	Yes (1)	High (9)
**10**	Lucre and Corten (2013) (7)	Yes (1)	Yes (1)	Yes (1)	Yes (1)	Yes (1)	Can’t tell (0.5)	Yes (1)	Can’t tell (0.5)	Yes (1)	Yes (1)	High (9.5)
**11**	Maynard et al. (2023) (12)	Yes (1)	Yes (1)	Yes (1)	Yes (1)	Yes (1)	Yes (1)	Yes (1)	Yes (1)	Yes (1)	Yes (1)	High (10)
**12**	Altavilla and Strudwick (2022) (46)	Yes (1)	Yes (1)	Yes (1)	Yes (1)	Yes (1)	Yes (1)	Yes (1)	Yes (1)	Yes (1)	Yes (1)	High (10)
	% of Included Studies Rated as Yes (1)	100%	100%	100%	66.66%	100%	33.33%	100%	83.33%	91.67%	100%	N/A
**Key**												
High (>8-10)												
Moderate (6-8)												
Low (≤5)												

### 3.3 Thematic synthesis

Five main themes with 13 sub-themes were developed during the synthesis process representing different aspects of participants’ experience of group CFT: 1) Participants’ experiences prior to the intervention, 2) initial response to the idea of participating in the CFT intervention, 3) participants’ experiences of the intervention: valued and beneficial aspects, 4) valued outcomes of the intervention, and 5) moving on from the intervention.

**Figure 2:**
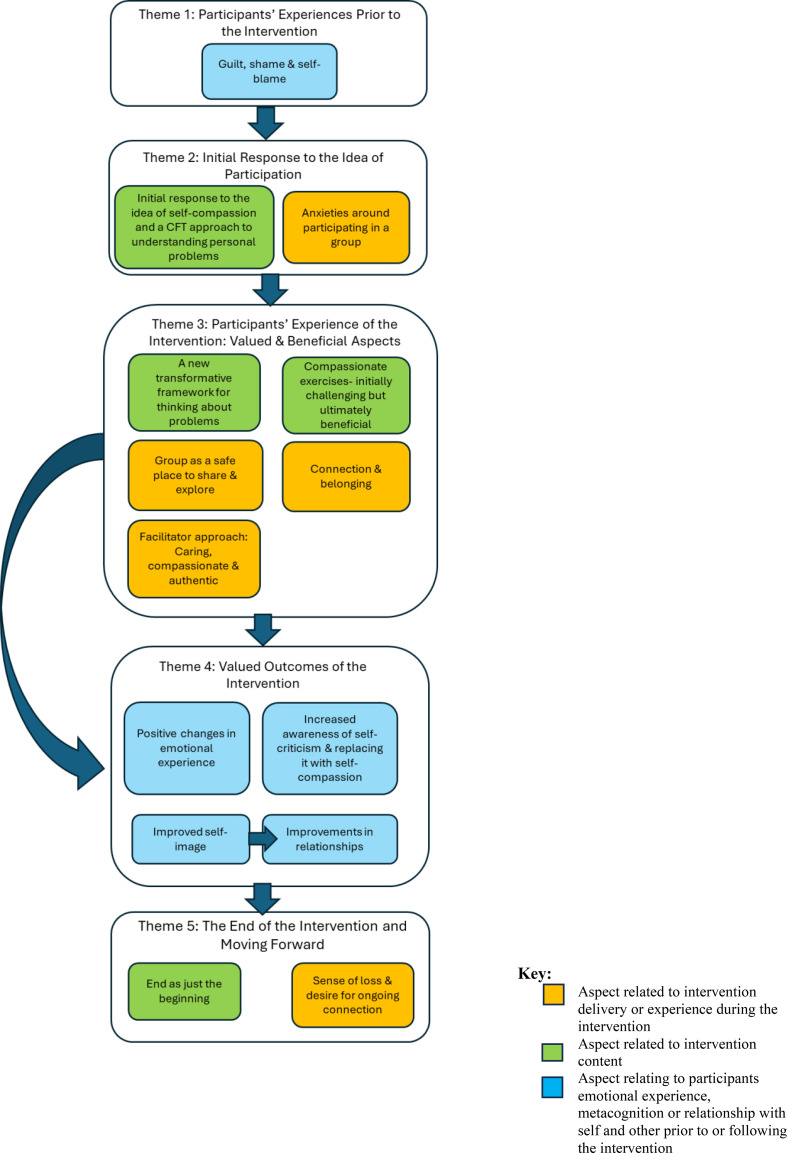
Diagram depicting themes and subthemes in the thematic synthesis*. *Organised according to participant journey through the intervention- pre-intervention (top) to post-intervention (bottom).

A matrix of themes is presented in **Table 5**, illustrating which themes were present in the 12 included studies. The themes and their relation to one another are depicted in **Figure 2**.

**Table 5:** Matrix of theme representation within the 12 included studies.

	Study: Authors and year	Theme 1: Participants’ experiences prior to the intervention: Guilt, shame and self-Blame	Theme 2: Initial Response to the Idea of Participation		Theme 3: Aspects valued or considered beneficial					Theme 4: Valued outcomes of the intervention				Theme 5: The end of the intervention and moving forward	
			Variation in initial response to the idea of self-compassion and a CFT approach to understanding personal problems	Anxieties around participating in a group intervention	The “Tricky Brain” and Three-Systems Model	Compassionate exercises: Initially challenging but ultimately useful	The group as a safe place to share and explore	Connection and belonging	Approach of facilitators: caring, compassionate and authentic	Positive changes in emotional experience	Increased awareness of self-criticism and replacing it with self-compassion	Improvements in self-image	Improvements in relationships outside of the group	Sense of loss and desire to maintain connections	The end ss just the beginning
1	Altavilla and Strudwick (2022) (46)	–	–	✓	✓	✓	✓	✓	–	✓	–	–	✓	✓	✓
2	Ashfield et al. (2021) (10)	✓	✓	✓	✓	–	✓	✓	✓	✓	✓	✓	✓	–	✓
3	Ashworth et al. (2015) (63)	✓	–	–	✓	–	✓	✓	✓	✓	✓	✓	✓	–	–
4	Clapton et al. (2018) (54)	–	–	–	✓	✓	✓	✓	–	✓	✓	✓	✓	–	–
5	Heriot-Maitland et al. (2014) (11)	–	–	–	✓	✓	✓	–	–	✓	✓	–	✓	–	–
6	Gilbert et al. (2022) (9)	–	✓	–	✓	✓	✓	✓	–	✓	✓	✓	✓	✓	✓
7	Goad and Parker (2021) (53)	–	–	–	✓	✓	✓	✓	✓	✓	✓	✓	✓	–	–
8	Gooding et al. (2020) (58)	✓	–	–	✓	✓	✓	✓	✓	✓	✓	–	–	✓	✓
9	Lucre and Corten (2013) (7)	–	✓	–	–	–	✓	✓	–	✓	✓	–	–	✓	✓
10	Maynard et al. (2023) (10)	✓	✓	–	–	✓	✓	✓	–	✓	✓	✓	✓	–	✓
11	Mullen et al. (2020) (59)	✓	✓	–	✓	–	✓	✓	✓	✓	✓	✓	✓	–	–
12	Raynor et al. (2022) (29)	✓	–	✓	–	✓	✓	✓	✓	✓	–	–	–	✓	–

✓ Theme identified; - Theme not identified.

#### 3.3.1 Theme: Participants’ experiences prior to the intervention: guilt, shame and self-blame

This theme related to participants’ thoughts and feelings about themselves and others prior to the intervention. In several studies (7, 46, 53, 54) individuals were identified as suitable candidates for CFT on the basis of having these kinds of thoughts and feelings.

Prior to taking part in CFT, participants talked about experiencing intense dislike towards themselves and high levels of shame, guilt, and self-blame for their mental health difficulties and the impact of these. As described by one participant: *“I deserved all that happened to me, so everything that’s happened I absolutely deserved it, that I’m pathetic, I’m a failure, that I’m weak … and everything that’s happened is pretty much my fault … it was the bottom line, you know it was, I deserved it, everything”* (10).

Some participants had had negative experiences of sharing their problems with others in the past, including with healthcare professionals (29). In these instances, the responses they received seemed to suggest to them that others were unable or unwilling to understand them or their difficulties, that they were judging them negatively for struggling, or that their difficulties were too much for other people: “*I think I have had a lot of professionals not just sort of therapists and that but teachers at schools who respond quite, aggressively worried … I almost felt like I was being told off”* (29).

Participants negative views of themselves and negative expectations about the potential for others to understand and support them, led them to self-isolate or feel disconnected from others: *“I just thought I was really weird … and it was like … I just wish I could flick a switch and be strong, and I wish I could get on with it, that’s all I want to do is get on with it but I couldn’t, so that made me feel really weird and because of that I think I was very hard on myself”* (10).

#### 3.3.2 Theme: initial response to the idea of participating in group CFT

Theme 2 contained two sub-themes that reflected participants’ initial responses to the idea of taking part in group CFT, when the idea was initially presented to them. These negative responses represent potential challenges to initial engagement with the intervention.

##### 3.3.2.1 Subtheme: variation in initial response to the idea of self-compassion and a CFT approach to understanding personal problems

Some participants reported responding negatively to the concept of self-compassion when it was first introduced. Some of these negative views included seeing self-compassion as self-indulgent or as a sign of weakness. Some participants associated self-compassion with undesirable characteristics such as arrogance, complacency, or laziness. Many participants viewed the idea of developing self-compassion with suspiciousness, scepticism, or worry: “I just associated self-compassion with being sort of like, Oh if I am being self-compassionate to myself I will become self-indulgent and cocky and lazy and erm become a bad person cause I will just—I will let myself go and I will lose sense of what’s right” (59).

Even if they could see the benefits of their being compassionate towards others, many participants harboured scepticism about the benefits of cultivating compassion towards themselves: *“I’m a compassionate person towards others but compassion towards me is a sign of weakness so I was very much, it’s not for me, so it’s for others and I can give it, but I don’t want it, I don’t want it from myself and I don’t want it from others” (* 10). However, participants also expressed being intrigued by the idea of self-compassion, and compassion more broadly, and expressed a desire to explore this further: “*to know a bit more of what other people think about caring, and what I think about caring”* (59). When participants were provided with more detailed information prior to the intervention, the authors reported that participants were very enthusiastic about the potential benefits of compassion and the helpfulness of the CFT framework for understanding their difficulties (9).

##### 3.3.2.2 Subtheme: anxieties about participating in a group

Participants expressed initial anxiety about the idea of taking part in a group intervention. A key fear for participants was being judged by other group members, which was highlighted by one participant who also described a fear that hearing other people’s experiences might trigger their own PTSD symptoms: *“People judging you … and also me hearing anything that happened for other people and whether it would trigger thoughts and memories and cause flashbacks really”* (10).

Anxiety of participating in a group intervention might have been contributed to by the assumption that the intervention would involve a lot of personal disclosure and discussion of difficult or traumatic personal experiences. This assumption, however, was mistaken: *“I thought it would be more, not intrusive, but like personal, that you would have to talk about the* sp*ecifics of your experiences, whereas it hasn’t been like that…”* (29).

#### 3.3.3 Theme: participants’ experiences of the intervention - aspects valued or considered beneficial

This theme related to aspects of the intervention that participants valued or believed to be beneficial. The aspects of content and delivery described in the five sub-themes reflect factors likely to have facilitated ongoing engagement with the intervention and factors that helped facilitate change for participants.

##### 3.3.3.1 Subtheme: the ‘tricky brain’ and three systems model - new and transformative framework for thinking about one’s problems

Psychoeducation on the ‘tricky brain’ and the three motivational and affect-regulation systems is a key part of CFT (2, 18, 37, 65). Across studies, participant’s reports suggested that they found the psychoeducational aspect of the intervention valuable. Specifically, their accounts suggested that they understood and found the material presented plausible, and that they were able to make use of what was presented to help them to better understand how their problems had developed and were maintained. The possible exception to this was participants with intellectual disabilities (53, 54); despite adaptations made to how the material was delivered, it was, as also noted by study authors, difficult to establish whether participants had been able to fully grasp the nuances of the material presented.

Within a CFT model, the experience of suffering is conceptualised as something common to all human beings and the product, largely, of factors beyond our control. As such, the CFT model offers individuals a de-stigmatising, de-personalising, and ‘*not my fault’* framework for understanding one’s difficulties. Across the studies, participants highlighted that CFT offered them a new way of conceptualising their difficulties, and that this made a significant impression on them. They reported that it reduced their sense of being the only one with these difficulties, as exemplified by this account: *“I really liked the sort of clinical psychology aspect of it … explaining to us how our brain works because then you don’t feel like it’s such a personal problem, it’s like, well, all humans have the same brains and this is why my brain has done that, and you don’t feel alone, you think ‘oh, I’m part of the human race then’ and this is how we all work”* (10). For many participants, the message that they were not to be blamed for their difficulties, was a realisation that was accompanied by strong emotions: “*And it had quite a profound effect on me when we were doing the meditation and he used the words, ‘it’s not your fault’, I had a huge weight lifted and it came out in, in tears…”* (9).

Ultimately, it was this reframing of their difficulties using the CFT model that seemed to enable participants to start thinking and feeling differently about themselves and their experiences and, eventually, to make better choices for themselves. Participants described how re-conceptualising their difficulties within a CFT framework instilled in them a new or renewed sense of responsibility and accountability for their own choices and decisions, which they found motivating: *“I have learned that no one else can do this for me, and that sucks sometimes, but I have to reassure myself because I wouldn’t believe someone else anyway”* (7).

##### 3.3.3.2 Subtheme: compassionate exercises - initially challenging but ultimately useful

Participants indicated that many found the compassionate exercises challenging initially. Reasons for this included self-criticism, difficulty imagining, disturbing images arising in the mind, feeling pressured or becoming preoccupied with getting it ‘right,’ and difficulty concentrating. Some participants found the exercises produced a more intense emotional experience than expected: *“I found it very emotional. A lot of the exercises brought up emotion, tears, and stuff like that, which I am quite comfortable with now because I know that’s just part of my way to process things. But it’s sometimes still quite surprising when you do the activities*” (9).

Most, however, were able to overcome these initial difficulties, eventually coming to find the compassionate exercises useful. Participants achieved this by reducing the pressure they put on themselves and by tailoring and modifying their approach to fit their cognitive profiles. For example, if concentration was the problem, the participant might reduce the length of the exercise. Making these adjustments enabled individuals to get more out of the practices and to integrate them into their daily lives: “*I do it* (compassionate mind practices) *in the real world as well now, quite easily. And the way I do it just by not making a big deal out of it, not expecting too much, not expecting magical things, but just getting clear in the mind*’ (9).

The reports of participants with intellectual disabilities (53, 54) suggested that they might find the experiential exercises in CFT easier to engage with than other aspects of the intervention: “*The exercises, like the breathing, easier to focus my mind than loads of words”* (53). However, they also indicated that some participants with intellectual disabilities may require more support to complete home practice of compassionate exercises.

##### 3.3.3.3 Subtheme: the group as a ‘safe space’ to share and explore

An aspect of the intervention that participants particularly valued was the group itself and their relationships with other group members, as highlighted by study authors using the following terms: “*A compassionate atmosphere’* (59), a “*psychological safeness”* (10), and an “*in-it-together-security”* (63).

Participants’ reports suggested that, over time, they came to experience the group as a place of safety where they could talk openly about their problems, be understood, and be sure of receiving a compassionate response: “*Everybody is open and just say anything, you know they are not going to judge you because they are in the same boat and everybody is just understanding, basically, you can just sort of say anything and you feel a bit safer”* (29), Having a safe space was described by participants as a great relief and was highly valued, particularly as, for some participants, this was not necessarily something they had experienced or had access to elsewhere. Over time, having access to this space appeared to erode the shame and sense of isolation participants experienced.

The sense of safety and security participants experienced in the group intervention benefited them in several ways. Firstly, as they trusted the group, they were able talk about personal experiences and problems that they had previously felt too shameful to disclose. In doing so it was possible, with the help of the group, for these problems and experiences to be examined, explored and problem solved. Secondly, hearing others talk about their similar experiences enabled some participants to put words to experiences of their own where they had not been able to previously and, in doing so, obtain more clarity around these: “*I think there has been a couple of times when someone else has said something and it’s just really made everything really, really clear”* (29). Being able to be fully open in sharing their difficulties made them seem more manageable for some participants- For example, one participant described how being able to use the term ‘self-harm’ openly “*takes the power away”* (29). Another benefit was that the group provided a safe base from which to explore the new strategies introduced as part of CFT and to benefit from these.

##### 3.3.3.4 Subtheme: connection and belonging

Over time, the repeated, reciprocal sharing of experiences between group members and having these received by other group members with recognition, understanding, and compassion built a deep sense of connection and belonging for participants within the group. Participants talked about how much they came to value their relationships with other group members and also with therapists/group facilitators: “*The group itself and the volunteers (facilitators) within the group were the apex of importance to me”* (58). This was the case even for participants with a diagnosis indicative of relational difficulties (e.g., participants with a personality disorder) and for participants whose relationships with others with the same disorder/difficulty may have previously been characterised by a level of competitiveness [e.g., individuals who self-harm (29) or those with eating disorders (59)]. Participant described that, over time, the group evolved into its own community based on mutual understanding and shared purpose. When group members encountered therapeutic challenges, they could draw on the strength and wisdom of the group: *“It did feel like we were a little bunch of warriors, which really kind of, it amped me up, it gave me the strength that I needed to push through that kind of big brick wall of denial”* (10).

##### 3.3.3.5 Subtheme: approach of facilitators - caring, compassionate and authentic

Participants’ comments suggested that they valued the approach taken by those facilitating the group. They appreciated that facilitators were caring, compassionate, and genuine, as well as being well-informed and credible. It is likely that the facilitator’s caring and compassionate approach served as a model for participants in their attempt to respond to themselves compassionately as well as increasing the feeling of safety that participants experienced in the group.

Part of the caring and compassionate approach to facilitation that participants appreciated was that they felt encouraged but not pressurised to contribute: “*The encouragement that we got and the whole approach of [the facilitators] was so compassionate … and it was so, it was done in such a caring way and a sensitive way that it just kind of left it wide open for you to engage with it. There was no barriers unless you put them up yourself … it was just so compassionately done; so I think probably what helped me the most”* (59).

Participants described facilitators as bringing more of their personhood and humanity to the role than they expected: “*I have not thought about it before but it’s like they* (the facilitators) *share an appropriate amount that doesn’t feel unprofessional, but it also feels like they’re not just a therapist, they are a person”* (29). This approach resulted in a flatter hierarchy and more even distribution of power in the group: “…*like, it doesn’t feel like it’s a therapist or two therapists sort of looking down and teaching. It is like a group, and everyone discusses what they want to discuss including the therapist and it feels like everybody is on the same level which I think is important. Like nobody, nobody comes across as like, ‘I am running it so today we are going to talk about what I want to talk about.’ There is none of that”* (29). This aspect of the facilitators’ approach increased participants’ sense of safety and security within the group, encouraging openness: “*Whereas a lot of therapists … actually just keep everything completely closed and I find that really hard because I am going in saying sort of my deepest darkest secrets … Whereas here it’s just like we are all, we are all in it together*” (29). This and other participant reflections suggest that the facilitators’ stance helped to reinforce notions of common humanity because their experience of affiliative emotions and compassionate flow within the group extended to include not only group members but also facilitators.

#### 3.3.4 Theme: valued outcomes of the intervention

This theme contained four sub-themes related to the positive changes participants perceived or reported as a result of taking part in the intervention. These changes included increased experiences of positive emotions, a reduction in and greater control over negative emotions, increased awareness of self-criticism and increased self-compassion, improvements in self-image, and changes in relationships.

##### 3.3.4.1 Subtheme: positive changes in emotional experience

Participants described positive changes in their emotional experience as a result of the intervention. As well as a reduction in negative emotions like shame and guilt, participants stated that they were experiencing more positive emotions, particularly those associated with the affiliative system (e.g., feelings of peace, contentment, closeness, and connection to others). Compassionate exercises such as soothing rhythm breathing appeared to be helpful for participants in helping them access calm and contented feelings, as suggested by the following exchange in which the interviewer asks about the impact of this breathing exercise for the participant: *“Interviewer: What would you say it’s changed? What has it helped you do? Participant:: Be the … inner peace of you … like, looking at yourself from the inside”* (54).

Participant reports suggested that not only did they experience a reduction in negative emotions such as shame, self-blame, and anxiety from taking part in the intervention, but they also felt better able to cope with these ‘negative’ emotions when they arose in daily life. Reasons for this seemed to be threefold: 1) the intervention gave participants new, concrete tools to use in stressful situations, 2) that CFT offered a different way of conceptualising these emotions, which enabled participants to develop a less antagonistic relationship with them and 3) drawing on the ‘three-systems’ model enabled participants when in difficulty to gain some distance and perspective from their emotions.

Regarding reason one, that CFT offered new tools to use in stressful situations, an exemplar of this is this participant who made use of the breathing exercises they learned on the course to help them manage anxious feelings: “*I close my eyes, and do the breathing exercises, and I find it helps me with me walk ‘cos I used to have panic attacks when I went out, but it don’t happen so much now”* (54). Having the compassionate exercises/practices as tools to help with emotion-regulation helped participants experience a greater sense of control and enabled more effective personal problem-solving: “*With the compassionate mind training, it’s much easier to cope. Because I can face a problem head-on and go from there. I don’t see many problems really, now. Now I’m able to sort them out now and concentrate and focus”* (63).

As previously described, the CFT intervention also helped participants to manage their emotions by suggesting a new way of conceptualising and relating to them. This way of understanding emotions was something that many of the participants were able to learn and use. From a CFT perspective, all emotions, even ‘negative’ ones like anger and fear, have evolved to serve a particular function or purpose. Being introduced to this perspective meant that participants were able to cultivate a less antagonistic relationship with these emotions. Participants reported that they were better able to tolerate these emotions without pushing them away, fighting with them or using unhealthy coping behaviours: “*So*, *I’ve kind of learnt now that you can’t get rid of anxiety, cause we actually need it, because it’s like in our DNA, it’s in our genetics and it’s part of evolution, so it’s more about having to go, okay, I’m going to have to sit with this anxiety and change the relationship with it and how I see it and that’s been a big thing for me”* (9). Participants were able to develop this ability to recognise and be ‘with’ their own distress – a recognised part of being self-compassionate and a necessary stepping stone to alleviating distress by taking compassionate action.

Participants’ experiences in the group might have reinforced the shift towards greater distress tolerance. Once psychological safety was established in the group, facilitators (and likely, later, other group members) supported participants in engaging with and expressing challenging emotions: *“*… *because coming here [to the group], I can get out*, *kind of, what I’ve been feeling and wanting to say and how sort of life has affected me”* (58).

In relation to reason three, participant reports also suggested that learning about the three-systems model in itself helped participants to respond to negative emotions as they arose. There was evidence that participants internalised the model and could draw upon it ‘in the moment’ to understand the emotions they were experiencing and to gain perspective and distance from them: “*So, it’s easier to deal with situations because, I recognise that there’s my brain function, I recognise that there’s my emotions at play, and the three systems. So, it gives me a choice now, it’s like I can see it quite objectively now, where before, I was just in it and I couldn’t see the wood for the trees…”* (9).

##### 3.3.4.2 Theme: increased awareness of self-criticism and replacing it with self-compassion

Participants reported being more aware of their self-criticism in the moment as a result of taking part in the intervention. As a result, they were better able to interrupt spirals of self-criticism and shame when they occurred: “*I catch myself out daily using the ‘I should have/could have/ought to have-type phrases and swiftly remind myself to ‘be compassionate!”* (7). When self-criticism arose in the mind, many participants talked about responding to it with a gentle reminder to themselves to be compassionate (as the participant in the previous quote describes). Others described dealing with self-critical thoughts by letting them pass by and not engaging with them: *“The voice in head telling me ‘I’m useless, wrong unwanted’- I used to tell it to go away or who do you think you are? But now I have stopped listening to it, I don’t hear it at all”* (7).

Participants talked about responding to themselves more compassionately following CFT, especially in times of difficulty. This shift towards being more self-compassionate was accompanied and enabled by a profound shift in participants’ attitudes towards self-compassion that occurred over the course of the intervention. From their original position of scepticism or suspicion, participants came to view self-compassion as positive, a source of strength and central to them achieving their personal goals: *“I used to be scared that I would be stuck on a compassionate sofa, going nowhere … I never realized that being kind to myself could help me DO things”* (7).

##### 3.3.4.3 Subtheme: improved self-image

Participants’ reports suggested that CFT helped them to make a positive shift in how they viewed themselves. As described in Theme 1, before the intervention, participants held highly negative opinions of themselves, seeing themselves as unworthy, inadequate, bad, and blameworthy. A key idea in CFT is that all humans are flawed, that all humans make mistakes, but all humans are, nevertheless, valuable and worthy of compassion (1, 18, 37). It appeared that CFT enabled participants to start the shift towards this more balanced and realistic view of themselves. For some participants, there was a re-appraisal of the role they played in past events: “*I had to realise that it’s not my fault … it was the other, the person who was in an adult mind and I was a child and so the blame is with them, it was nothing to do with me, I think that was one of the biggest moments”* (10).

Some participants indicated that taking part in CFT had led them to discover or rediscover their authentic selves where previously they had been unable to extricate their true selves from their emotions, critical inner voice, or diagnosis. One participant talked about “*finding the person you really are … on the inside”* (54). This clearer sense of self helped participants feel clearer about their needs and values, and more in control of their emotions and actions, resulting in greater self-confidence and self-belief.

Holding a more balanced view of the self, and a compassionate orientation helped individuals to make more positive choices for themselves, as reflected in the following quote: *“So, the compassion that I give to myself now is that accountability. If you go and* sp*end that 50 in wherever, what have you got to do afterwards? … Is this the compassionate thing to do? It’s the indulgent thing to do, without a doubt, but is this a compassionate thing? So, it’s that questionable thing I have all the time, it’s almost given me my conscience back a little bit. So much so that I’ve lost two-and-a-half kilos [laughs] just purely by going, ‘Who do you want to be? Your compassionate image’. I look at that and I go, ‘That’s who I want to be now’”* (9). Like this participant, other participants also talked about using their compassionate image to guide how they would ideally wish to be with themselves.

##### 3.3.4.4 Subtheme: improvements in relationships

In addition to the significant changes reported by participants in how they perceived and related to themselves, participants also reported significant changes in how they perceived and related to others as a result of CFT participation: “*In terms of like friendships, some really good ones this year where I am just a lot more open and willing to admit vulnerabilities and erm yeah that’s been really good”* (59). These changes could have been prompted by individuals’ re-appraising their own worth, as well as their experience of compassionate interactions with group members and facilitators. The safe, containing environment of the group enabled participants to experiment with being vulnerable and their authentic selves with others; the compassionate responses that they then received and the deep connections they formed over time may have reinforced the importance of this in other relationships. It is also possible that they provided a helpful template for what to aim for or expect in relationships with others. Participants’ experiences of sharing and listening to one another’s experiences in the group, of feeling understood by other group members, and of recognising elements of their own experience in other’s stories seems to have generated a sense of commonality that went beyond the group, suggesting the possibility of greater connection with those outside of it, and reducing anxiety/threat around this.

Participant accounts suggested that they felt more confident in their existing relationships and also more confident in forming new relationships after taking part in the intervention: *“It’s massively improved my relationship with friends … trying to make new friends was something I was terrified of before … I have less anxiety about that now because I feel confident in my ability to handle it and I feel confident that I’m perhaps someone that someone might want to know which before was just I had very little faith in people finding me interesting”* (10). Participant accounts also suggested a re-prioritising of themselves and their needs in their relationships with others. Previously, participants reported that they were likely to put others’ needs first before their own. However, following CFT, they were much more likely to treat their needs as being on an equal footing: *“I have started caring more about myself and what I want out of life and not thinking so much about others- not saying that I don’t care about others, because I do, but I have started to put myself first now, not others”* (63).

Following CFT, participants also reported being more likely to turn to others for emotional support when faced with challenging situations: “*…a skill I learn is that if I’m feeling very distressed whereas before I might just have dived into food … [In a challenging situation] I instead rang my fiancé and told him and we had a chat, and that sort of eased the situation”* (59). These changes in relationships outside the group could have contributed to by the positive experience participants had of being supported and responded to compassionately when they shared their difficulties and challenges in the group.

Participants also said they understood and empathised with others more readily and were more likely to respond compassionately to others outside the group than before the intervention, things that, over time, are likely to lead to improvements in relationships: *“My daughter, she’s 4 years old now. Obviously she’s at a stage now where she’s learning everything new. So by me showing her I love her, care her, care for her, not by just buying her stuff, I talk to her, be her ear, so when she comes up from nursery, ask her ‘how was your day?’ maybe, you know, just like that, things like that, simple things. About my partner as well, ask him how was his day, is he okay, does he need to talk about anything, is he upset, have you eaten, you know, simple things like that. It makes life … it’s nice. It’s just nice to have that somebody ask you that”* (11).

#### 3.3.5 Theme: the end of the intervention and moving forward

This theme contained two sub-themes relating to different aspects of participants’ experiences of the end of the intervention and the thoughts and feelings they had around moving forward.

##### 3.3.5.1 Subtheme: sense of loss and desire to maintain connections

Participants felt sad and a sense of loss about the intervention ending. Some participants also expressed feelings of anxiety. Anxious feelings related to whether they could sustain or build on the changes they had made without regular meetings with and support from the group. This feeling of loss reflected the powerful connections that had developed between group members over the course of the intervention and the value group members placed on these relationships: *“I know it sounds really weird … I have not actually thought about finishing the group, because it’s been like a proper little support network where it’s going to be really weird not coming, even though I have to be dragged up the stairs every single week; it’s going to be the first Thursday and be like … even though we have got our little packs and stuff to focus on, there is no people even though we have got tutors, there is no people”* (29). Many participants talked about wanting to keep the group and the relationships they had formed going after the intervention ended.

##### 3.3.5.2 Theme: the end as just the beginning

Participants tended to see completing the CFT intervention as just the start of a much longer journey toward dealing with their difficulties and developing self-compassion: *“I still feel like there’s a lot of change to come with me … because I do feel like the full benefit of the course hasn’t come to fruition at all I just feel like this is the tip of the iceberg’* (10). This quote, from another participant, also speaks to a sense of there still being much change to come, but also a sense of direction and hope as a result of taking part in the intervention: *“I left each group feeling a little better in myself, a little scared but feeling for one I had somewhere to go and what ‘moving on’ really meant”* (7).

## 4 Discussion

This metasynthesis of 12 studies explored the experience of individuals with psychological difficulties taking part in group CFT, with a view to identifying issues pertinent to the acceptability of the intervention, and factors promoting engagement and retention. Across diverse samples it was possible to identify many common themes in participant experiences that spoke to the acceptability of this intervention as well as factors that facilitated or could hinder engagement and retention of participants. The review provides novel insights into the interaction between participant experiences and CFT content and its delivery. It also presents a comprehensive understanding of the factors most important to consider in the planning and delivery of group CFT.

The findings suggest a high level of acceptability of group CFT across clinically diverse groups, both in terms of content and delivery. The content of the intervention made sense for and was useful to both male and female participants, helping them to reframe their difficulties in a way that reduced the guilt and shame that they had been struggling with and their sense of isolation from others. There was evidence that participants were able to integrate the learning from the group into day-to-day life; they reported being more aware of their self-critical thoughts and emotions, being able to link this to the three motivational systems. This awareness helped them gain perspective on their mental processes, meaning they were then able to make better decisions for themselves, including in times of difficulty, something that they found extremely valuable.

The overall positivity of participant feedback indicates a high level of acceptability of both the content of compassion-focused interventions and of the way in which it was delivered, supporting Craig et al.’s (4) conclusions.

It was notable that, although participants were initially anxious about taking part in group CFT, they did, over the course of the intervention, come to value this aspect of intervention delivery. This is demonstrated in their reports of a real sense of loss of the connections they had made with other group members and their anxiety around whether they would be able to sustain the therapeutic gains they had made without the support of the group when the intervention came to an end. Participants valued the group as a safe psychological place to discuss their mental health difficulties, found comfort through identifying shared experiences, and offered support to each other through therapeutic challenges. These kinds of benefits of the group format are very similar to those described in other qualitative studies of group-based psychotherapeutic interventions in individuals with mental health difficulties (66). However, as suggested by the authors of several of the included studies (10), it is possible that the group format might have particular therapeutic benefits, especially in CFT. Group processes have the potential to have a powerful reinforcing effect for the key concepts and messages of CFT (e.g., common humanity, the value of turning towards suffering and offering compassion). Individuals could potentially gain from having more opportunities to experience the direct, ‘felt’ experience of compassion when CFT is delivered in a group. This experiential knowledge could supplement the intellectual understanding that participants gained from the psychoeducational components of the intervention in a way that enhances therapeutic benefits. The felt experience of community and solidarity within the group might help to reinforce the concept of common humanity more than is possible when the intervention is delivered one to one and therefore have more power to erode the sense of shame and isolation individuals experienced. The experience of compassionate relating with multiple others provided in a group (as opposed to the one relationship in one-to-one delivery) could also have a more powerful effect on an individual’s relationship with themselves and relationships with others outside of the group, the relationships between group members acting as a model for how to relate in an authentic, genuine and compassionate way. Furthermore, in a group, individuals could plausibly gain more of the direct experience of the positive emotions associated with activating the affiliative system in response to the compassion offered by facilitators and other group members. In addition to strengthening the affiliative system, this experience might also reinforce the importance of self-compassion, making individuals more receptive to the idea that it may be beneficial, whilst also providing more opportunities to learn how to respond compassionately through seeing this modelled. Together with being an efficient way to deliver therapy within services, offering CFT in a group format might also lead to better outcomes and a better experience for service users.

An interesting finding of the synthesis relates to the participants’ positive experience of the CFT group facilitators (Theme 3.5). Participants had mentioned finding it helpful that facilitators were more open to sharing their own experiences and struggles than perhaps they had expected or had experienced in previous therapy sessions. Participants talked about how this emphasis on the commonality of their experience with facilitators increased their sense of safety in the group, helping them to be more open about their difficulties. When delivering CFT, therapists are encouraged to embody and apply its key principles (67). As such, in CFT, there seems to be more of emphasis on interacting with and responding to clients in a caring and compassionate manner. Therapists are also encouraged to be more ‘human’ or ‘authentic’ in the therapeutic relationship. Findings from this synthesis suggested that participants responded positively to this approach from facilitators.

### 4.1 Strengths, limitations and future research

Strengths of the current review included its use of rigorous methods including a systematic search, quality assessment of included papers and inter-rater reliability checks. Searches were not limited by language or date of publication. Although the methodological rigour in the conduct of this review is an evident strength, some limitations should be acknowledged. We included only studies published in peer-reviewed journals. By excluding non-peer-reviewed literature and the wider grey literature, it is possible that we introduced location and publication biases, so some caution is advised when transferring findings. This review also concentrated on the experiences of participants undertaking CFT and as such did not include the perspectives of clinicians or commissioners. Few studies have so far been published that used qualitative methods to explore clinician or commissioner perspectives of CFT, so this is an important area for future research.

In recent years there has been an attempt to gain more conceptual clarity around the concept of acceptability and its component parts as it pertains to healthcare interventions. On the basis of a systematic review of reviews that claimed to define, theorise or measure the concept, and also consideration of potentially relevant theories from health psychology and behaviour change, Sekhorn et al. (68) generated the *Theoretical Framework of Acceptability* (TFA). This framework, which is comprised of seven domains, when used to inform the development of topic guides and the analysis of primary qualitative data (e.g., from interviews and focus groups), has the potential to offer a broader and richer understanding of the acceptability of particular healthcare intervention. Future studies using the TFA to inform study design and analysis may have the potential to add additional insights to our current understanding of the acceptability of CFT.

As for any review, the comprehensiveness of the final synthesis is impacted by the current state of the literature in this field. It is notable that none of the included qualitative studies reported on the experiences and views of those who decided not to participate in the intervention, or who dropped out during the intervention. This is not unusual in this type of research, given the difficulties inherent in recruiting those who chose not to take-up or drop out of interventions into research studies. However, future qualitative research focusing on the perspectives of individuals who do not take up the offer of intervention, or who commence the intervention but drop out, would be valuable to further developing our understanding of reasons for intervention attrition or drop-out.

While our search was not limited to studies published in English, or studies published in particular countries, it is noteworthy that included studies came from only three countries. Given this lack of cultural diversity in the included samples, future studies are clearly indicated to examine CFT’s cross-cultural acceptability.

None of the included studies were part of funded randomised controlled trials in which participants might have been offered incentives for participation or in which the intervention might be offered in a form in which it would not ordinarily be offered in NHS services in the UK. This is noteworthy as it suggests that the findings of the included studies, and, by implication, of this metasynthesis, are truly representative of the acceptability of the intervention, delivered within clinical settings.

### 4.2 Clinical implications

The current review highlights key aspects relevant to the content and delivery of CFT to individuals with psychological difficulties. Clinical recommendations and implications for maximising engagement and retention in CFT for this participant group (individuals with psychological difficulties) are presented below (**Table 6**). These recommendations are derived from the findings of the synthesis and are, therefore, grounded in qualitative data and driven by the voices of participants themselves.

**Table 6:** Suggested clinical recommendations and implications.

Acceptability-related outcome/goal	Recommendation
**Engagement**	• Clinicians to be mindful of potential negative attitudes and beliefs about self-compassion in initial discussions with service users. Questionnaire measures, such as the Gilbert, McEwan, Matos and Rivers (69), may be useful for eliciting these beliefs which can then be explored and normalised. • Clinicians to also be aware of the potential for anxiety around participating in group therapy in this client group. Presenting information on the potential benefits of the group may help to alleviate these anxieties, as well as providing reassurance that the focus of the group will not be on revealing a lot of personal detail.
**Retention**	• Clinicians to be mindful that service users may initially find compassionate exercises difficult and that they may need additional support to help problem-solve and individually tailor compassionate exercises to fit their cognitive profile and lifestyle. • CFT interventions offered should include all the recognised components as outlined by Gilbert (2, 18, 20), i.e. psychoeducation, compassionate exercises, and group discussion as all appear valuable to participants. • It is likely to be necessary to make adaptations to the delivery of the intervention content, particularly the psychoeducational material, when working with individuals with intellectual disability and other forms of neurodivergence. • Facilitator approach is important to the participant experience of CFT. A good standard of training and supervision will support facilitators to maintain the level of openness and responsiveness that participants value. Personal practice of CFT may also be also help support group facilitators in this (70).
**Other**	• If feasible, it may be helpful to offer additional ‘top up’ sessions for participants to help sustain and build on therapeutic gains. • Group facilitators should ensure that they offer support to group members to process the feelings of loss they may experience at the end of the intervention, and to think about how and where they can access support from others following the intervention. • If appropriate, it may be useful to help group participants to consider how they may stay in contact following the group so that group members can continue to offer each other peer-support. • When evaluating group CFT interventions services should ideally include a longer-term follow-up, so that benefits that might have accrued following the intervention are not missed.

### 4.3 Conclusion

This was the first review to comprehensively gather and synthesise qualitative studies exploring the experiences of group CFT for people with psychological or mental health difficulties. Our aim in doing so was to establish a more detailed and nuanced understanding of the acceptability of CFT for this client group. Findings indicated that these individuals found taking part in the intervention to be a positive experience overall which resulted in beneficial changes in areas that mattered to them, including making it easier for them to manage their emotions, to improve their self-image, and to function better in their relationships with others. The importance of facilitator approach and group processes in facilitating engagement and change were emphasised in participants’ accounts.

## Footnotes

1 Gilbert P, Petrocchi N, Kirby J. Compassion focused therapy therapist manual. Compassionate Mind Foundation.
